# Pinobanksin from peony seed husk: A flavonoid with the potential to inhibit the proliferation of SH‐SY5Y

**DOI:** 10.1002/fsn3.3786

**Published:** 2023-11-15

**Authors:** Wen‐Tao Chen, Ying‐Yang Zhang, Qiang Qiang, Lin‐Ling Zou, Ping Zou, Ying Xu

**Affiliations:** ^1^ School of Biological and Food Engineering Changzhou University Changzhou Jiangsu China; ^2^ Changzhou Wujin No. 3 People's Hospital Changzhou Jiangsu China

**Keywords:** C18 isolation, molecular dynamics, pharmacophore screening, podophyllotoxin

## Abstract

Pinobanksin, as one of the flavonoids, has powerful biological activities but has been under‐recognized. In this study, we optimized the extraction method of phragmites from peony seed shells by using organic solvent extraction. The yield of PSMS was 10.54 ± 0.13% under the conditions of ethanol volume fraction 70%, extraction temperature 70°C, material–liquid ratio 1:25 g/mL, and extraction time 60 min; the optimized PSMS could be effectively separated in S‐8 macroporous resin coupled with C18. The relative content of PSMS was increased from 0.42% in PSMS to 92.53% after C18 purification; the antioxidant activity test revealed that pinobanksin could exert antioxidant ability by binding catalase (CAT) enzyme. Second, it was found that pinobanksin could effectively inhibit the proliferation of SH‐SY5Y cells, mainly by binding to BCL2‐associated X (BAX), B‐cell lymphoma‐2 (BCL‐2), and cyclin‐dependent Kinase 4/6 (CDK4/6) to produce more hydrogen bonds to inhibit their activities. This study confirms the medicinal potential of pinobanksin and provides the basis for the proper understanding of pinobanksin and the development of related products.

## Graphical abstract

Optimization of extraction conditions of flavonoids (PSMS) from peony seed shells. The relative content of pinobanksin was elevated from 0.42% in PSMS to 92.53% after C18 purification. The inhibitory effect of pinobanksin on the proliferation of SH‐SY5Y cancer cells is discussed in the context of molecular dynamics. Target protein screening of pinobanksin by pharmacophore method.

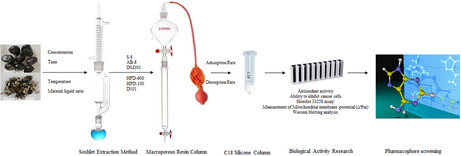

## 1 INTRODUCTION

In agrifood production, a significant wastage of resources occurs due to the underutilization of valuable bioactive compounds, including flavonoids, phenolic compounds, and anthocyanins (Rana et al., 2021). This issue is rooted in the broader context of sustainable agriculture and resource optimization (Treml & Šmejkal, 2016), where the efficient extraction and application of these bioactive compounds could have profound implications for both economic and environmental sustainability (Horowitz & Gentili, 1960). Extensive research in the field of bioactive compounds has highlighted their potential in various applications, from pharmaceuticals to functional foods, emphasizing the need for a more systematic approach to their extraction and utilization in agrifood production (Bangar et al., 2022).

Flavonoids, as a subset of these bioactive compounds, have garnered significant attention in recent years (Chen et al., 2019). They are secondary metabolites found in various plant species and have demonstrated a wide range of health‐promoting properties, including antioxidant effects and potential benefits for cardiovascular health (Bisol et al., 2020). Understanding the diversity of flavonoid structures and their biological activities is crucial in harnessing their full potential in agrifood production. Previous studies have explored the structural variations among flavonoid compounds and their relationships with specific health benefits (Gouveia et al., 2022), providing a foundation for further investigations into their extraction and utilization from agricultural by‐products.

Efficient separation and purification techniques are essential in the extraction of bioactive compounds (Deng et al., 2022) from agricultural waste materials. Macroporous resin (Shen et al., 2022) has emerged as a promising choice due to its favorable adsorption characteristics, cost‐effectiveness, and ease of operation. This resin's ability to selectively capture flavonoids from complex matrices, such as peony seed shells, underscores its significance in optimizing resource utilization within the agrifood industry. Moreover, the utilization of C18 silica gel columns (Zhang et al., 2009) in conjunction with macroporous resin further enhances the efficiency of flavonoid separation. These innovative techniques build upon prior research in chromatography and resin‐based separation methods (Martinenghi et al., 2020), offering a more sustainable approach to extracting valuable compounds from agricultural waste.

The importance of biomolecular complexes and their role in fundamental biological processes cannot be overstated. Research in this area has expanded our understanding of how molecules interact within biological systems. Recent advances in computational biology and drug discovery (Meuwly, 2019) have enabled researchers to explore protein–small molecule interactions (Scafuri et al., 2021), protein–peptide (Hu et al., 2022) interactions, and protein–protein (Singh et al., 2018) interactions with greater precision. The integration of computational tools, such as molecular docking and pharmacophore modeling (Bajusz et al., 2021), has expedited drug development processes and holds immense potential for the discovery of novel bioactive compounds. Previous studies have paved the way for the application of these computational techniques in the investigation of pinobanksin's binding to key proteins, shedding light on its potential therapeutic properties (Xiong et al., 2022).

**FIGURE 1:**
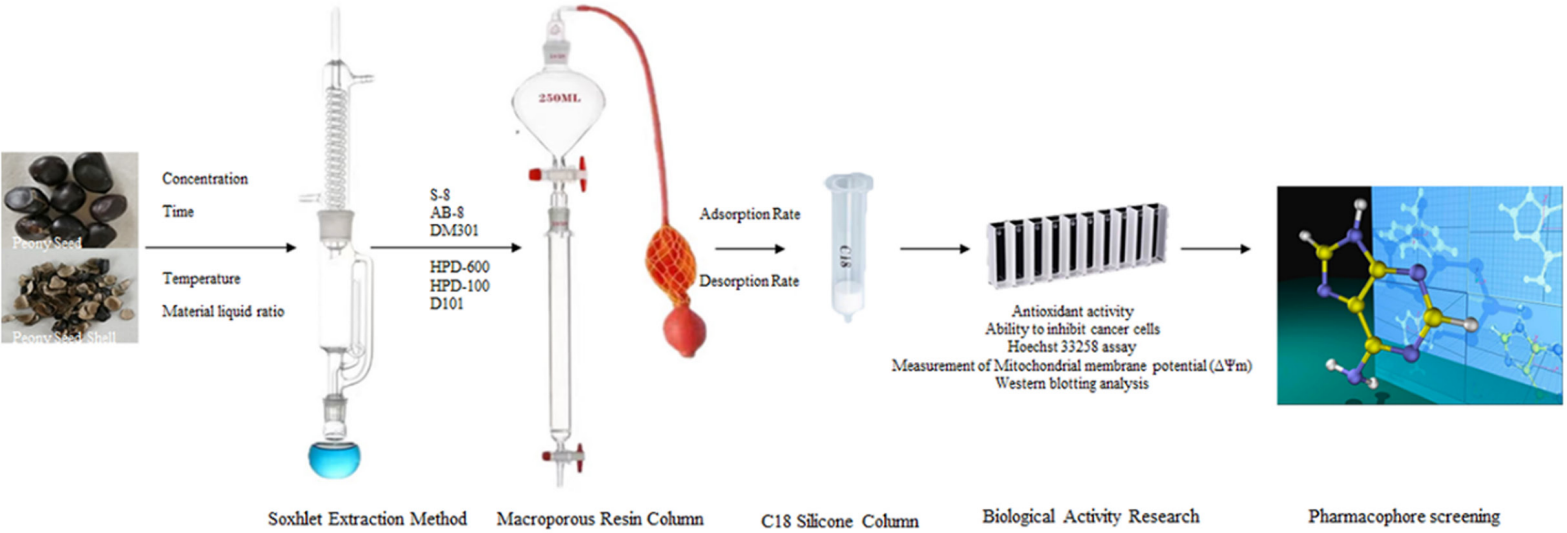
PSMS technology route.

Pinobanksin, a flavonoid present in various natural sources, has demonstrated a myriad of biological activities in prior research. These activities span from antioxidant effects (Zheng et al., 2018) to its role in inhibiting specific cellular processes (Bang & Ahn, 2021). Building upon this foundation, our experiment focused on the extraction and characterization of pinobanksin from peony seed shells (Liu et al., 2018). In this experiment (Figure 1), by employing advanced separation techniques and biological assays, we aimed to establish a more comprehensive understanding of its potential applications in cancer cell proliferation inhibition. The results of our pharmacophore screening and molecular docking studies provide valuable insights into the molecular mechanisms underlying pinobanksin's interactions with target proteins, presenting a promising avenue for further research in drug development.

## 2 MATERIAL AND REAGENTS

1,1‐Diphenyl‐2‐trinitrophenylhydrazine (DPPH), ascorbic acid, o‐diazafil, hydrochloric acid, nitro tetrazolium chloride (NBT), reduced nicotinamide adenine dinucleotide (NADH), and 5‐methyl phenazine sulfate methyl ester (PMS): analytical purity, Sinopharm Chemical Reagent Co.

UV–visible near‐infrared spectrophotometer: UV‐3600, Shimadzu Enterprise Management (China) Co., Ltd; precision electronic balance: ATY224, Changzhou Wantai Balance Instrument Co., Ltd; high‐throughput tissue disintegrator: Tissuelyser‐48, Shanghai Jingxin Industrial Development Co. Scientific; UPLC: Acquity, Waters; vortex mixer: XH‐T, Baita Xinbao Instrument Factory, Jintan District; and nitrogen purge instrument: Reacti‐thermo, Thermo Fisher Scientific.

## 3 EXPERIMENTAL METHODS

### 3.1 PSMS extraction

The Peony seed meal shell (PSMS) was crushed into powder by crusher passed through an 80‐mesh sieve, and set aside. Weigh 1.0 g PSMS powder, change the extraction time to 30–150 min, the volume fraction of ethanol 50–90%, the extraction temperature 50–90°C, change the material–liquid ratio (m PSMS: V ethanol) 1:20–1:40 g/mL; and carry out single‐factor experiments. The values of the factor levels of response surface experiments were derived from the single‐factor experiments; the extraction time, extraction temperature, ethanol concentration, and liquid‐to‐material ratio were selected as the influencing factors; the amount of flavonoid extraction was used as the response value; and the surface response experiments were conducted on peony seed shells using Design‐Expret 8.0.6 software (Ma et al., 2016), respectively, and the response surface factor table is shown in Table 1.

**TABLE 1:** Response surface test factor levels.

Level	A ethanol volume fraction (%)	B extraction temperature (°C)	C material–liquid ratio (g/mL)	D withdrawal time (min)
−1	60	60	1:20	30
0	70	70	1:25	60
1	80	80	1:30	90

### 3.2 PSMS content determination

#### 3.2.1 Standard curve plotting

The standard curve of rutin (González‐De‐Peredo et al., 2022) was plotted according to the method described in the literature, and its precision, reproducibility, and spiked recovery were determined to examine the accuracy of the curve.

#### 3.2.2 Determination of PSMS content and yield

Accurately pipette 1 mL of flavonoid extract into a 10 mL volumetric flask, dilute with 4 mL of 30% ethanol (v/v), add 4 mL of 1% AlCl_3_ solution, and fix the volume with 30% ethanol. Shake well and let stand for 10 min, and measure the absorbance value at A415 nm (Yang et al., 2012).

The total flavonoid yield of peony seed hulls was calculated according to the equation.

$$\text{PSMS yield}\left(\%\right)=\frac{c\cdot V_{2}\cdot V_{1}}{V_{3}\cdot m}\times 100\%$$

Where:

*c*—mass concentration of total flavonoids in peony seed shell, μg/mL;

*m*—a mass of the weighted sample, μg;

*V*_1_—the volume of the extraction solution, mL;

*V*_2_—the volume of the reaction system at the time of determination, mL;

*V*_3_—the volume of aspirate at the time of determination, mL.

### 3.3 PSMS separation and purification

#### 3.3.1 Parameters of the macroporous resin

Different properties of macroporous resins have different separation effects for substances with different polarities, and the differences in adsorption and desorption rates of six different macroporous resins for flavonoids were comprehensively compared to screen the resins. The parameters of different resins are shown in Table 2.

**TABLE 2:** Comparison of the properties of six different macroporous resins.

Resin model	Characteristic	Specific surface area (m^2^/g)	Application
S‐8	Polarity	120–150	Yang et al. (2012)
AB‐8	Weak polarity	490–550	Xi et al. (2015)
DM301	Medium polarity	330–380	He et al. (2017)
HPD‐600	Polar	550–600	Wang et al. (2021)
HPD‐100	Nonpolar	650–700	Yin et al. (2019)
D101	Nonpolar	500–550	Pan et al. (2017)

#### 3.3.2 Calculation of adsorption rate

A total of 2.5 g from six macroporous resin solutions of AB‐8, D101, S‐8, X‐S, HPD‐500, and NKA‐9 were taken out and put into a conical flask, 25 mL of PSMS solution was added and placed in a constant temperature shaking chamber for 8 h (25°C, 180 rpm/min), the supernatant was removed, and the remaining PSMS concentration in the solution was calculated. The adsorption rate can be calculated by the following equation method (Lv et al., 2018).

$$E\left(\%\right)=\frac{A_{1}-A_{2}}{A_{1}}\times 100\%$$

where *A*_1_ is the PSMS content of the solution before adsorption, mg/mL; and *A*_2_ is the remaining PSMS content of the solution after adsorption, mg/mL.

#### 3.3.3 Calculation of desorption rate

The macroporous resin solution was put into a conical flask, 25 mL of 75% ethanol (v/v) was added, it was put into a constant temperature oscillator for 8 h of full absorption at a constant temperature of 25°C and 180 rpm/min, and finally, the supernatant was taken to determine the remaining PSMS content in the solution, which is because ethanol can compete with the molecular state products in the macroporous resin solution for adsorption causing the products to dissolve in ethanol, thus resolving PSMS from the macroporous resin solution (He et al., 2017).

$$D\;\left(\%\right)=\frac{A_{3}\times V_{1}}{\left(A_{1}-A_{2}\right)\times V_{2}}\times 100\%$$

where *A*_1_ is the PSMS content in the solution before adsorption, mg/mL; *A*_2_ is the remaining PSMS content in the solution after adsorption, mg/mL; *A*_3_ is the PSMS content in the solution after desorption, mg/mL; *V*_1_ is the volume of desorbed liquid, mL; and *V*_2_ is the volume of adsorbed liquid, mL.

#### 3.3.4 Adsorption kinetics

The adsorption kinetics study was carried out in an oscillating incubator at 25 rpm/min at 30°C. UV analysis was performed on samples from six different resins every 10 min from 0 to 120 min, respectively. The isothermal adsorption curves of the quasi‐primary kinetic model and quasi‐secondary kinetic model of the resins were plotted using the concentration of PSMS in the above clear solution as the horizontal coordinate and the adsorption amount at adsorption equilibrium as the vertical coordinate. Meanwhile, the corresponding isothermal adsorption equations were obtained using the Langmuir and Freundlich adsorption models.

The quasi‐primary kinetic equation was (Wang et al., 2021)

$$\ln\left(Q_{e}-Q_{t}\right)=\ln Q_{e}-K_{1}\times t$$

The quasi‐secondary kinetic equation is given by (Lv et al., 2018)

$$Q_{t}=\frac{{q_{e}}^{2}\times k_{2}\times t}{q_{e}\times k_{2}\times t+1}$$

where *Q*_e_ represents the absorption of the resin at PSMS absorption stabilization (mg/g); *Q*_t_ represents the absorption of the resin at time *t* (mg/g), while *K*_1_ and *K*_2_ are kinetic parameters.

Langmuir isothermal adsorption equation is given by (Lv et al., 2018)

$$\frac{C_{e}}{Q_{e}}=\frac{1}{Q_{m}\times K_{a}}+\frac{C_{e}}{Q_{m}}$$

The Freundlich isothermal adsorption equation is given by (Shen et al., 2022)

$$\ln\left(Q_{e}\right)=\frac{1}{n}\ln C_{e}+\ln K_{b}$$

where *C*_e_ denotes the PSMS content in the discharge solution after absorption stabilization (mg/L); *Q*_e_ denotes the absorption amount at absorption stabilization (mg/L); *Q*_m_ denotes the maximum adsorption amount (mg/L); *K*_a_ is the magnitude of the driving force between the macroporous epoxy resin and PSMS in the Langmuir equation; and *K*_b_ is the coefficient related to the adsorption amount of macroporous resin in the Freundlich equation.

#### 3.3.5 Determination of dynamic elution process

##### Determine the optimal flow rate of the sample

A 30 × 300 mm column was selected and installed vertically, and the column was loaded by the wet method. In the experiment, flow rates of 1, 2, 3, 4, and 5 BV/h were selected for collection. After adsorption on the macroporous resin for 30 min, 75% ethanol (v/v) was added and 1 mL was collected in each tube. Then, the PSMS content was calculated according to the method in 4.3.2, and the curve was plotted to determine the optimal loading flow rate (Yin et al., 2019).

##### Determination of optimal loading sample volume

The optimal loading rate was used to start the collection, and 1 mL was collected per tube to measure the PSMS content and depict the leakage curve to determine the best loading volume for the best results.

##### Determine the optimal ethanol elution concentration

By controlling the optimal loading volume and flow rate, and eluting with different concentrations of ethanol (v/v), 1 mL of each tube was collected, the PSMS content was calculated, and the curve was plotted to determine the best elution concentration (Pan et al., 2017).

##### Determine the best elution rate

The experimental elution flow rate was 0.25, 0.5, 0.75, 1.0, 1.5, and 2.0 mL/min for elution. One milliliter was collected from each tube, the PSMS content was measured, and the curve was plotted to determine the optimal elution rate.

##### Separation by macroporous resin

The adsorption separation was carried out according to the optimal loading volume, optimal loading flow rate, optimal ethanol elution concentration, and optimal elution speed, and the different components after separation were tested for their antioxidant capacity.

#### 3.3.6 C18 column separation

The C18 spherical silica gel column was selected from Changzhou Santai Technology Company with a particle size of 15 mm and a pore size of 100 Å (Table 3). The mobile phase was 70% acetonitrile containing 0.1% TFA (v/v), and the components with good antioxidant effects after separation by macroporous resin were selected to be sampled. The antioxidant capacity was tested.

**TABLE 3:** Properties of C18 spherical silica columns.

Projects	Test results	Testing method
Column size	20 g	–
Maximum working pressure	400 psi (27.5 bar)	–
pH range	2.0–8.0	–
Specific surface area (m^2^/g)	312	Multipoint BET method
Pore size (mL/g)	0.88	Multipoint BET method
Particle size distribution D50 (mm)	15.2	Particle size analyzer
Carbon content (wt/%)	16.3	Elemental Analysis

### 3.4 Activity test

#### 3.4.1 Antioxidant activity

(1) According to the method of Fan (Lv et al., 2018) with slight modifications. Crude PSMS solutions were prepared at mass concentrations of 20–120 μg/mL, respectively, with GSH as the control group. Two milliliter of 0.2 mmol/L DPPH radical solution (dissolved in 95% ethanol) was added to 2 mL of samples, mixed, reacted at room temperature, protected from light for 30 min, and the absorbance of the samples was measured at A517 nm. The blank was 2 mL sample + 2 mL 95% ethanol, and the control was 2 mL DPPH +2 mL 95% ethanol. The results were determined using the following equation:

$$\text{DPPH clear activity}\;\left(\%\right)=\frac{\left(A_{b}-A_{s}\right)}{A_{b}}\times 100\%$$

where *A*_s_ and *A*_b_ represent the absorbance of the sample and blank, respectively.

(2) The method of Fan (Chen & Zhang, 2014) was referenced. The mixture consisted of 4 mL of 1,10‐o‐phenanthroline (5 μmol/L) and 4 mL of FeSO_4_ (5 μmol/L); then, 3 mL of phosphate buffer (pH = 7.4) was added, followed by 3 mL of H_2_O_2_ (0.01%) and 4 mL of sample (20–120 μg/mL). Finally, the mixture was left at 36°C for 1 h and the absorbance was measured at A536 nm. Control: distilled water was used instead of PSMS solution, and other reagents were the same as the samples. Blank: distilled water was used instead of H_2_O_2_, and other reagents were the same as the samples. The results were determined using the following equation:

$$\mathrm{OH}\;\text{clear activity}\;\left(\%\right)=\frac{\left(A_{s}-A_{c}\right)}{\left(A_{b}-A_{c}\right)}\times 100\%$$

where *A*_s_, *A*_c_, and *A*_b_ represent the absorbance of sample, control, and blank, respectively.

(3) Referring to the method of Wang (Fan et al., 2017) with slight modifications. As follows: sterile water was configured with mass concentrations of 20–120 μg/mL for samples with GSH control. 1.5 mL of sample was added sequentially with 0.5 mL 300 μmol/L NBT (pH 8.0 Tris–HCl buffer configuration), 0.5 mL 468 μmol/L NADH (pH 8.0 Tris–HCl buffer), and 0.5 mL 60 μmol/L PMS (pH = 8.0 Tris–HCl buffer), shock mixing, water bath at 25°C for 5 min, light absorption value was measured at A560 nm, and the buffer was used as a blank control instead of the sample.·O_2_^−^ radical scavenging rate was calculated according to the following formula:

$$O_{2}^{-}\;\text{clear activity}\left(\%\right)=\left(1-\frac{A_{s}}{A_{b}}\right)\times 100\%$$

where *A*_s_ and *A*_b_ represent the sample and blank absorbance, respectively.

(4) This was performed according to the method described by Wu (Wang et al., 2022). ABTS^+^ radicals were obtained by mixing 5 mL of 7 μmol/L ABTS solution with 88 μL of 140 μmol/L potassium persulfate solution at 20°C for 20 h. The absorbance at A734 nm was 0.70 ± 0.02 by adding about three times 75% ethanol (v/v). The sample consisted of 9.8 mL of diluted ABTS^+^ radical solution and 0.2 mL of 20–120 μg/mL sample. A mixture of 0.2 mL of distilled water and 9.8 mL of diluted ABTS^+^ radical solution was used as a blank, and 1 mg/mL of crude PSMS 0.2 and 9.8 mL of distilled water was used as a control. All mixtures were left at room temperature for 0.5 h and measured spectrophotometrically at A734 nm. The formula for the radical scavenging activity of ABTS^+^ was as follows:

$$\text{ABTS}+\text{clear activity}\;\left(\%\right)=\frac{\left[A_{b}-\left(A_{s}-A_{c}\right)\right]}{A_{b}}\times 100\%$$

where *A*_s_, *A*_c_, and *A*_b_ denote the absorbance of sample, control, and blank, respectively.

(5) Detection of PC12 cell viability by MTT method

A density of 1 × 10^5^ cells/mL was removed from PC12 bacteria and injected directly into 96‐well bacterial culture plates; after 24 h of pre‐cultivation, the medium was aspirated, 100 μL of serum‐free culture medium containing 25 μmol/L BPH was added, and control and blank groups were set up with four replicate experiments in each group. After 24 h of BPH treatment, 10 μL of MTT lysate was added to each well and incubated continuously for 4 h. Then, the supernatant was aspirated and 100 μL of formazan lysate was added to each well, mixed well, and put back into the bacterial incubator for up to 4 h until no purple crystals could be observed under an inverted microscope. The absorbance of each well at A570 nm wavelength was measured by an enzyme marker and the corresponding survival rate equation was expressed.

The survival rate equation is as follows.

$$\text{Survival rate}\;\left(\%\right)=\frac{A_{0}-A_{1}}{A_{2}-A_{1}}\times 100\%$$

where *A*_0_, *A*_1_, and *A*_2_ represent the OD of the experimental group, the OD of the blank group, and the OD of the control group, respectively.

(6) Determination of intracellular ROS content

To determine the intracellular ROS content according to the method of Zhu (Wu & Huang, 2017), the results of the intracellular ROS level in PC12 cells after PSMS treatment can be calculated by the following equation:

$$\text{Relative fluorescence intensity}\;\left(\%\right)=\frac{A_{0}-A_{1}}{A_{2}-A_{1}}\times 100\%$$

where *A*_0_, *A*_1_, and *A*_2_ represent the OD of the experimental group, the OD of the blank group, and the OD of the control group, respectively.

(7) Intracellular antioxidant activity (CAA) assay

The logarithmic growth phase cells were removed from PC12 cells, their density was adjusted to 6 × 10^4^ cells/mL, and then they were evenly dispersed in sterile all‐black 96‐well plates. After the bacterial cultivation was completed, the old cultivation medium in the wells should be removed, and sterile D‐Hank's aqueous solution was used for one to two rinses to ensure that the insufficiently adhered and dead bacteria were effectively removed. For comparison, 100 μL and DCFH‐DA probes were added to the experimental group, and 100 μL of diluted DCFH‐DA probes were added to the control and blank groups, respectively, for a more in‐depth study. The bacteria were incubated in a 37°C, 5% CO_2_ incubator for 1 h; rinsed with sterile D‐Hank's aqueous solution 2–3 times; 100 mL of ABAP aqueous solution (600 μmol/L) and 100 μL of sterile D‐Hank's aqueous solution were added to the test and control groups, respectively; followed by rapid placement into a fluorescence enzyme marker set at 5 min intervals and detected in real‐time for 1 h to obtain the true fluorescence information. The excitation wavelength was up to A485 nm, while the emission wavelength was up to A538 nm. The CAA values were calculated as follows:

$$\mathrm{CAA}\left(\%\right)=\left(1-\frac{S_{A}}{C_{A}}\right)\times 100\%$$

where *S*_A_ is the area of the fluorescence value versus the time formation curve after the addition of BPH; and *C*_A_ is the area of the fluorescence value versus the time formation curve for the blank group.

(8) Molecular docking

The PDB database was downloaded for superoxide dismutase (SOD) (PDB ID: 1E9O), CAT (PDB ID; 3QJ4), POD (PDB ID; 1M9Q), and GPX (PDB ID; 6ELW). The protein structures were imported into AutoDocktools (Zhu et al., 2022) (v1.5.6) for hydrogenation, charge calculation, charge assignment, assigning atom types, and saving in “pdbqt” format using Pymol 2.3.0 software to remove protein crystalline water, raw ligands, etc. Protein binding sites were predicted using POCASA 1.1 and docked using AutoDock Vina1.1.2, with SOD‐related parameters set to center‐*x* = 16.3, center‐*y* = 28.4, center‐*z* = 74.9; search space: size‐*x*: 50, size: 50, and size‐*z*: 50 (the spacing of each grid point is 0.375 Å); exhaustiveness: 10; and the rest parameters are set by default.

#### 3.4.2 Ability to inhibit cancer cells

Cells (SH‐SY5Y) were maintained in an incubator containing 5% CO_2_ and 95% air at 37°C. First, cells at a density of 3 × 10^5^ were inoculated in triplicate per well into 96‐well culture plates and incubated overnight. Various concentrations of all molecules were added to the cells and incubated for 24, 48, and 72 h. Cells were then treated with 10 μL MTT solution (5 mg/mL) and further incubated for 4 h. Subsequently, each well was treated with 100 μL 10% SDS‐HCl and incubated overnight at 37°C or washed, and then 100 μL DMSO was added. Finally, absorbance at A570 nm and A630 nm were recorded.

Different concentrations of compound 4f were added to SH‐SY5Y cells. Forty‐eight hours later, the cells were washed twice with PBS. Subsequently, cells were stained in the dark using 500 μL of DAPI (4 μg/mL) and membrane‐linked protein V‐FITC/PI (5 μL). Fifteen minutes later, cell cycle or apoptosis was analyzed by flow cytometry (Beckman Coulter) using CXP analysis software.

#### 3.4.3 Hoechst 33258 assay

SH‐SY5Y cells at a density of 7 × 10^5^ were inoculated cell/well into 24‐well culture plates and incubated for 48 h in the presence of compound 4F (1, 2, and 4 μmol/L) and PTL (4 μmol/L). Subsequently, the cells were stained with 1 μg/mL of Hoechst 33258 for 15 min in the dark. After 15 min, the cells were washed twice with PBS. Finally, the morphological changes were observed using a 20× objective fluorescence microscope.

#### 3.4.4 Measurement of mitochondrial membrane potential (ΔΨm)

SH‐SY5Y cells at a density of 7 × 10^5^ cells/well were inoculated into six‐well culture plates and incubated in the presence of compound 4F (1, 2, 4 μmol/L) and PTL (4 μmol/L). After 24 h, cells were washed twice with PBS. Subsequently, rhodamine 123 (5 μg/mL) was added to the cells and incubated for 30 min at 37°C, followed by two washes with PBS. All samples were analyzed by flow cytometry using CXP analysis software.

#### 3.4.5 Western blotting analysis

SH‐SY5Y cells at a density of 7 × 10^5^ were inoculated per well into six‐well culture plates. SH‐SY5Y cells were incubated with compounds 4F (1, 2, and 4 μmol/L) and PTL (4 μmol/L) afterward. The protein was fractionated on SDS‐polyacrylamide gel electrophoresis. Next, it was electrically transferred to PVDF membranes. All membranes were sealed in TBST buffer with 5% skim milk for 1 h and then incubated with primary antibodies (BCL‐2, BAX, CDK2/4/6, and β‐actin) for 4 h at 12°C. After washing for 1 h, the blots were incubated with a secondary antibody for 1 h. After washing again with TBST for 1 h, proteins were visualized using the ECL assay kit purchased from the Biyang Institute of Biotechnology (China).

#### 3.4.6 Molecular dynamics

All‐atom molecular dynamics simulations were performed separately based on the small molecules and protein complexes obtained by docking as described above as the initial structures, and the simulations were performed using AMBER 18 software (Trott & Olson, 2010).

The binding free energies between proteins and ligands were calculated by the MM/GBSA method for all systems. The long‐time molecular dynamics simulations may not be conducive to the accuracy of MM/GBSA calculations, so the MD trajectories of 45–50 ns were used as calculations in this study with the following equations (Salomon‐Ferrer et al., 2013):

$${\Delta G}_{\text{bind}}={\Delta G}_{\text{complex}}–\left({\Delta G}_{\text{receptor}}+{\Delta G}_{\text{ligand}}\right)$$

$$={\Delta E}_{\text{internal}}+{\Delta E}_{\mathrm{VDW}}+{\Delta E}_{\text{elec}}+\Delta G_{\mathrm{GB}}+{\Delta G}_{\mathrm{SA}}$$

In the equation, 〖Δ*E*〗_internal represents internal energy, 〖Δ*E*〗_VDW represents van der Waals interaction, and 〖Δ*E*〗_elec represents electrostatic interaction. The internal energy includes the bond energy (Ebond), angular energy (Eangle), and torsion energy (Etorsion); 〖Δ*G*〗_GB and 〖Δ*G*〗_GA are collectively referred to as the solvation‐free energy. Among them, GGB is the polar solvation‐free energy and GSA is the nonpolar solvation‐free energy. For 〖Δ*G*〗_GB, the GB model developed by Nguyen et al. is used for calculation (igb = 2). The nonpolar solvation‐free energy (ΔGSA) is calculated based on the product of surface tension (*γ*) and solvent‐accessible surface area (SA), ΔGSA = 0.0072 × ΔSASA. The entropy change is neglected in this study due to the high computational resources and low precision. The entropy variation was neglected in this study due to high computational resources and low precision.

### 3.5 Pharmacophore screening

To discover the potential proteins of the target molecules, we screened the target compounds against the pharmacophore library. Short molecules were constructed using the Maestro module of the Schrodinger 2021‐1 package. The pharmacophore library was selected from the library recently reported by Aurélien et al. (Hou et al., 2011), which was constructed based on the latest PDB eutectic molecules and contains 16,646 pharmacophore models. The potential acting proteins of the target compounds can be fully explored.

In this study, the phase module of the Schrodinger 2021‐1 package (Moumbock et al., 2021) was used for the target screening work. Considering the large number of rotatable bonds, we set the generated conformations of the target molecule to 200 during the screening process, so that the conformational space of the target molecule can be explored as much as possible and all pharmacophores of the defined protein can be considered as hit proteins when all pharmacophores are bound. The target proteins are further filtered after the screening based on the screening scoring fitness as well as manual selection.

### 3.6 Data processing

GraphPad Prism 5 Software (GraphPad Software, San Diego, CA, USA) was used for the analysis of the correlations between different parameters.

## 4 RESULTS AND DISCUSSION

### 4.1 Extraction process of PSMS

In accordance with the method described in Section 3.2.1, we employed the absorbance value at A510 nm as the vertical coordinate and the mass concentration of rutin standard as the horizontal coordinate. Data analysis and linear fitting were performed using Origin 2022b software to generate the rutin standard curve. The linear regression equation was found to be *y* = 0.0279*x* + 0.0166, with an *R* ^2^ = .99597. In this equation, ‘*y*’ represents the absorbance value, and ‘*x*’ represents the mass concentration. These results demonstrate that the flavonoid solution exhibits excellent linearity within the range 0–50 μg/mL, making it suitable for determining the mass concentration of flavonoid compounds.

**FIGURE 2:**
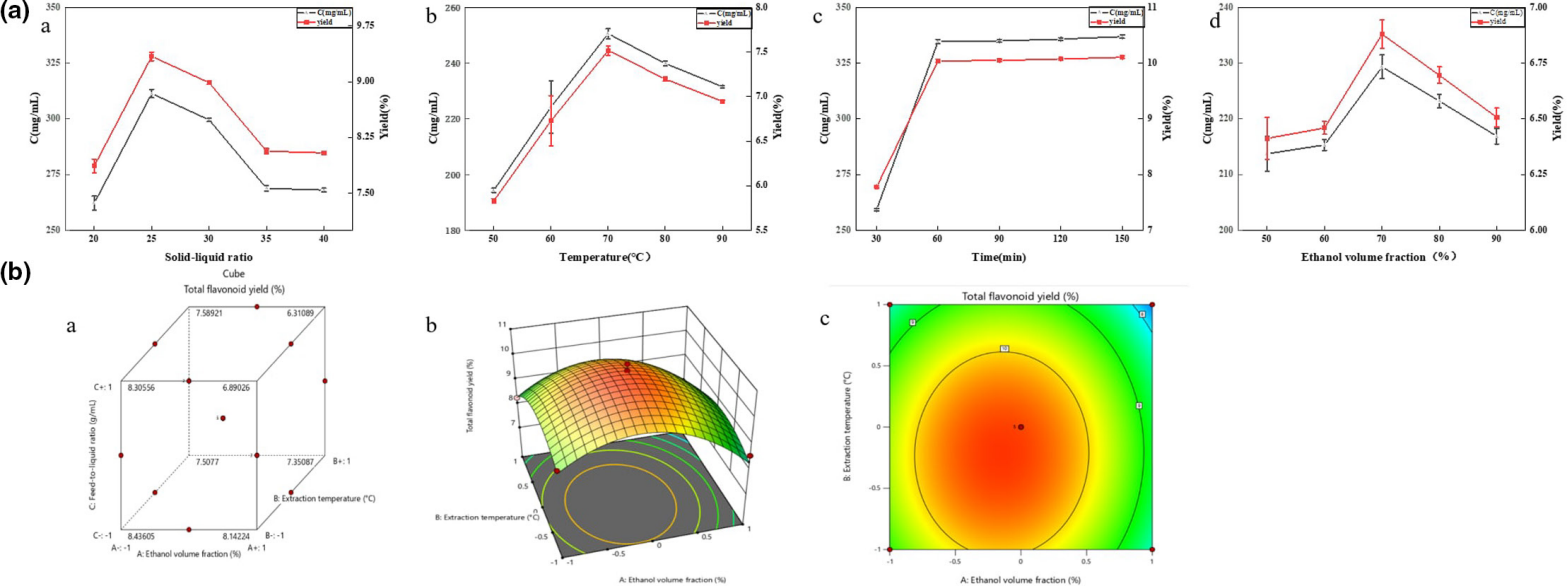
Optimization of PSMS extraction process.

As depicted in Figure 2A(a), when the material–liquid ratio was less than 1:25 g/mL, the total flavonoid yield from peony seed shells remained low. This can be attributed to the limited contact area between the peony seed shell powder and the extraction solvent, resulting in a lower extraction of total flavonoids. Conversely, when the material–liquid ratio exceeded or equaled 1:25 g/mL, the total flavonoid yield reached equilibrium. This suggests that the total flavonoid dissolution rate reached its maximum when the material–liquid ratio reached 1:25 g/mL. Therefore, the optimal feed–liquid ratio was determined to be 1:25 g/mL.

As illustrated in Figure 2A(b), extraction temperatures below 70°C led to a significant increase in total flavonoid yield from peony seed hulls as the temperature increased. This indicated that higher temperatures accelerated molecular movement, reduced solvent viscosity, and facilitated the transfer of flavonoids from the plant matrix through cell membranes. However, it is worth noting that some flavonoids are sensitive to high temperatures (Dixon et al., 2006). Consequently, when the extraction temperature exceeded 70°C, the total flavonoid yield from peony seed shells gradually decreased due to the decomposition of less stable flavonoids. In conclusion, the optimal extraction temperature was determined to be 70°C.

Moving to Figure 2A(c), the extraction rate of PSMS increased progressively with longer extraction times, up to 60 min. Beyond this point, there was no significant change as flavonoids reached saturation during the extended extraction period.

In Figure 2A(d), it is evident that the PSMS yield increased with the ethanol volume fraction. However, when the ethanol volume fraction exceeded 70%, the total flavonoid yield decreased. This reduction was attributed to the higher ethanol volume fraction promoting the dissolution of other nonflavonoid substances, thereby affecting the dissolution of PSMS (Dixon et al., 2006). Hence, the optimal volume fraction of ethanol as an extraction solvent was set at 70%.

The regression model (Figure 2B(a–c)) was analyzed using Design‐Expert 13 software, as shown in Figure 2B(a). The optimal conditions for PSMS extraction were determined to be 69.8% ethanol by volume, 69.7°C extraction temperature, 1:25.4 g/mL material‐to‐liquid ratio, and 59.8 min of extraction time. The model calculated the total flavonoid yield from peony seed hulls under these conditions to be 10.65%. To validate these results, we adjusted the optimal conditions to 70% ethanol by volume, 70°C extraction temperature, 1:25 g/mL material‐to‐liquid ratio, and 60 min of extraction time. The experiment was repeated three times, yielding a PSMS yield of 10.54 ± 0.13% (*n* = 3), which closely matched the predicted results. These optimized process conditions, derived from the response surface model, are accurate and reliable, serving as a valuable reference for subsequent practical operations.

### 4.2 Pinobanksin separation

**FIGURE 3:**
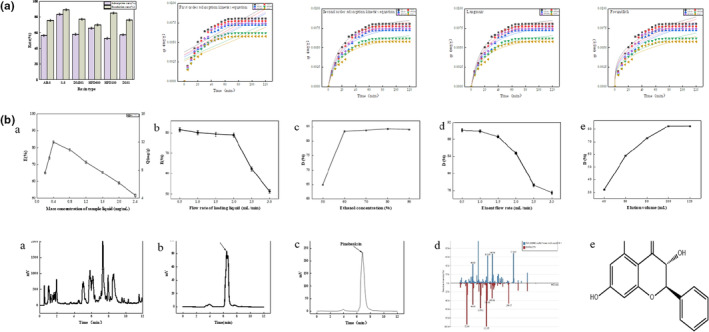
Pinobanksin isolation.

The adsorption and desorption rates are vital indicators for evaluating the separation efficiency of macroporous resins. Testing the adsorption and desorption rates of six different macroporous resins revealed that S‐8 macroporous resin, as shown in Figure 3A(a), achieved impressive rates of 83.47 and 89.54%, respectively. This success can be attributed to the resin's polar, reticular structure, and high specific surface area, which enable it to effectively screen the separation of compounds with varying molecular sizes. While particle propagation kinetic models may not capture every detail of the resin adsorption process, they provide insights into specific stages of absorption. Resin adsorption can be categorized into three distinct regions (Erşan et al., 2018): (a) the outer region, which is nearly saturated with absorption; (b) the middle region, where absorption occurs over a specific time; and (c) the innermost region, where absorption has not yet occurred.

As the adsorption process continues, the saturated zone expands, contributing to the diffusion process within the particle. The kinetic curve of the resin is illustrated in Figure 3A(a,b). It is noteworthy that the adsorption rate of all six resins is remarkably fast, reaching saturation within 120 min. Among these resins, S‐8 outperforms the others, with the adsorption capacity ranking as follows: S‐8 > AB‐8 > DM301 > HPD‐600 > HPD100 > D101. Based on these results, AB‐8 resin stands out as an efficient and cost‐effective option for PSMS separation. After careful consideration, S‐8 was chosen for the PSMS separation process.

The separation ability of macroporous resin is influenced by various factors, including loading speed, loading volume, eluent concentration, and elution flow rate. As demonstrated in Figure 3B(a), sample mass concentration significantly impacts the separation efficiency. The adsorption rate declined as the sample concentration increased, indicating that the macroporous resin reached saturation, causing the sample to be flushed out of the column before complete absorption. When the sample flow rate ranged from 0.5 to 2.0 mL/min, the adsorption rate of S‐8 macroporous resin remained stable at around 80%. However, at flow rates exceeding 2.0 mL/min, the adsorption rate dropped abruptly, likely due to rapid sample passage through the resin, resulting in inadequate adsorption time.

Figure 3B(c) highlights that the desorption rate (v/v) of PSMS eluted by a 60% ethanol–aqueous solution stands at 83.29%, significantly surpassing other ethanol concentrations. Consequently, 60% ethanol–aqueous solution (v/v) was selected as the eluent. The elution rate also plays a pivotal role in elution effectiveness. If the elution rate is too fast, PSMS molecules may fail to elute from the column. Referring to the elution curve, Figure 3B(d) demonstrates that the resolution curve (D) of the resin to PSMS remains relatively flat when the elution rate ranges from 0.5 to 1.5 mL/min. This may be attributed to the small elution rate and wake phenomenon when multiple components are simultaneously eluted. However, when the elution rate exceeds 1.5 mL/min, the resolution significantly decreases, compromising the separation efficiency. Hence, an elution flow rate of 1.5 mL/min is deemed appropriate.

To enhance separation efficiency, we studied the dynamic desorption effect of S‐8 macroporous resin concerning the eluent ethanol volume. As depicted in Figure 3B(e), the desorption rate of S‐8 macroporous resin gradually increased with an increase in eluent volume. When the eluent volume exceeded 40 BV and approached equilibrium, the desorption rate reached 82.46%. Therefore, an eluent volume of 40 BV was established as the optimal condition.

Since S‐8 macroporous resin typically does not separate individual components, we performed further separation using a preparative column (C18 spherical silica column). This column boasts high separation efficiency and can effectively purify mixtures into single components. It is widely employed for the purification of biological macromolecule compounds. The C18 column fraction underwent further separation, and its structure was analyzed using a Thermo Ultra‐High‐Performance Liquid System (Vanquish, USA) with a Waters HSS T3 (100*2.1 mm, 1.8 mm) liquid chromatographic column. The injection volume was 2 mL, and the column temperature was set at 40°C. Mobile phase A consisted of 0.1% formic acid–acetonitrile, while mobile phase B comprised 0.1% formic acid–water.

As shown in Figure 3C(a–c), the combination of S‐8 and C18 columns, along with liquid–mass analysis (Figure 3C(d) compared with the standard), successfully separated shortleaf pinene. The peak for shortleaf pinene appeared at 6.816 min with a mass‐to‐nucleus ratio of 272.07 and a C_15_H_12_O_5_ structure. Comparing peak areas (Figure 3C(c)), the relative content of shortleaf pinene increased from 0.42% in PSMS to 92.53% after C18 purification.

### 4.3 Exploration of pinobanksin activity

The antioxidant properties of flavonoid compounds are largely determined by their chemical structure (Oancea et al., 2017). Hydroxyl, peroxy, peroxynitrite, and other free radicals tend to interact with the hydroxyl groups on the B‐ring of flavonoid compounds, converting them into potent flavonoid radicals and initiating subsequent reactions. The number of hydroxyl groups on the b ring and the location of its substituents often determine the antioxidant capacity of flavonoid compounds. Free radicals are molecules or molecular fragments with unpaired electrons in their outer orbitals, making them highly reactive and prone to catalyzing harmful oxidative reactions with cellular proteins, lipids, or DNA. This oxidative stress can lead to impaired cellular function. Flavonoids, belonging to the extensive phenolic compound group, exhibit robust antioxidant capabilities, as they can neutralize free radicals and prevent their excessive accumulation (Andrade et al., 2018).

**FIGURE 4:**
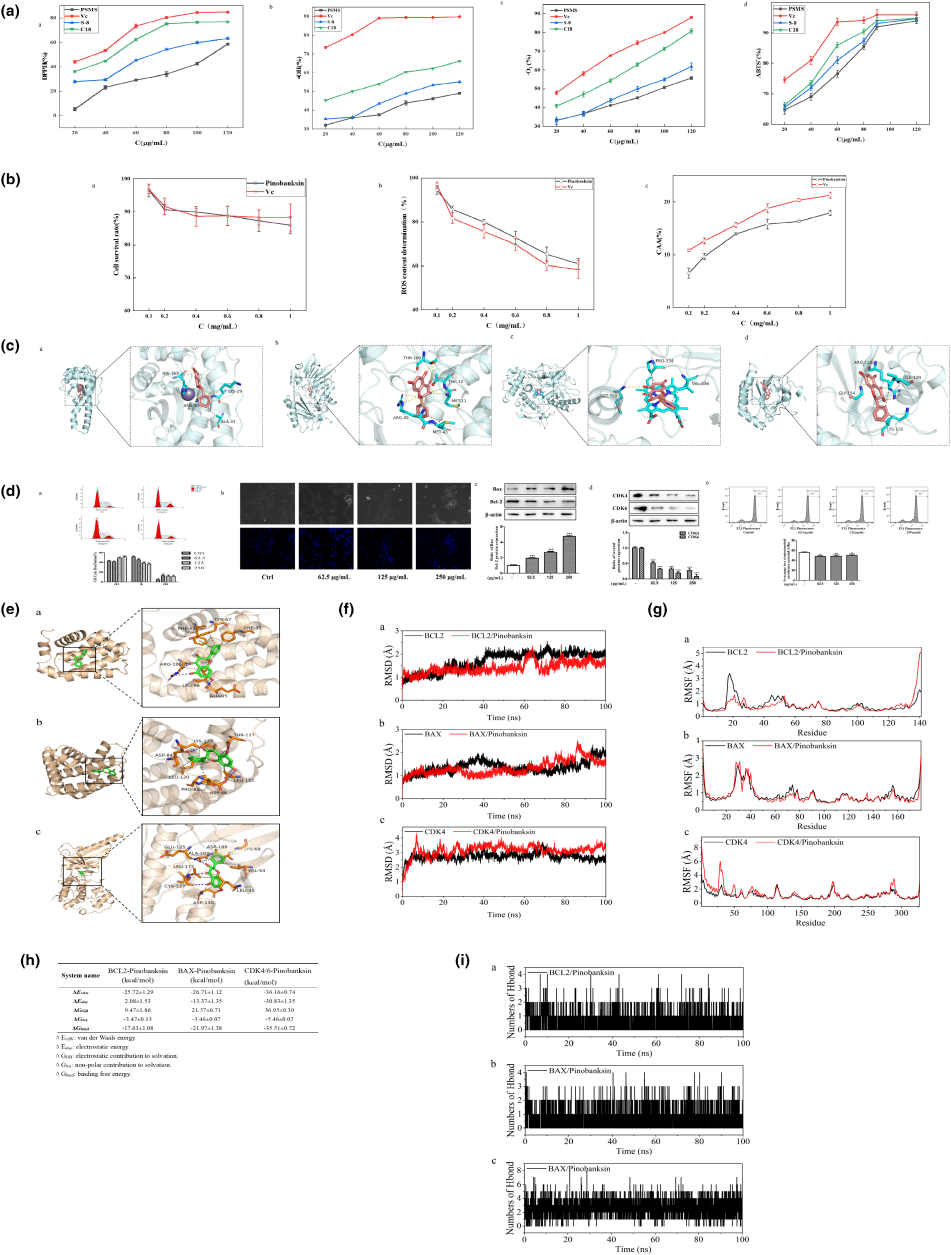
Pinobanksin activity investigation.

The scavenging rates of PSMS, S‐8 macroporous resin isolate, and Pinobanksin (C18 isolate) for DPPH radicals, ·OH radicals, ·O_2_^−^ radicals, and ABTS^+^ radicals are illustrated in Figure 4A(a–d), with Vc included at the same concentration for comparison. The scavenging ability of each sample increases progressively with the sample's mass concentration. Notably, the antioxidant capacity of PSMS, when purified by the C18 column, surpasses that in a mixed state with other flavonoids. This phenomenon might be attributed to the interactions between phenolic hydroxyl groups in the flavonoid structure, which can weaken the antioxidant capacity.

To assess the impact of pinobanksin concentrations ranging from 0.1 to 1.0 mg/mL on PC12 cell viability, the MTT method revealed that after 24 h of treatment, the survival rate of PC12 cells exceeded 85% (Figure 4B(a)). This finding suggests that pinobanksin, within this experimental range, does not exhibit significant toxicity. Reactive oxygen species (ROS), a natural by‐product of oxygen, play essential roles in cell signaling and organismal homeostasis. However, under conditions of heightened environmental stress (e.g., UV light or heat exposure), ROS levels surge (Ravisankar et al., 2020), potentially leading to severe damage to cellular structures and triggering oxidative stress. Measurement of ROS content at pinobanksin concentrations ranging from 0.1 to 1.0 mg/mL (Figure 4B(b)) indicated a reduction in ROS levels following pinobanksin addition, with a 31.82% reduction at a 1 mg/mL concentration, showcasing pinobanksin's intracellular antioxidant effect.

To further explore its mechanisms, we investigated its effects on Catalase (CAT) and cellular antioxidant activity (CAA). Primary mouse hepatocytes exhibited reduced cell viability under CAA exposure, and intracellular CAT activity showed a bell‐shaped response to CAA exposure, partially influenced by molecular CAT activity. Testing CAA activity at pinobanksin concentrations ranging from 0.1 to 1.0 mg/mL (Figure 4B(c)) revealed an upward trend in CAA activity with increasing pinobanksin concentration, with a 10.39% increase at 1 mg/mL of pinobanksin. These results confirmed pinobanksin's ability to enhance CAA activity and reduce ROS.

The antioxidant capacity of organisms typically results from the combined action of small molecules with antioxidant capacity and enzymes such as superoxide dismutase (SOD), CAT, peroxidase (POD), and glutathione peroxidase (GPX). To assess pinobanksin's antioxidant capacity, we employed molecular docking to analyze its interaction with SOD, CAT, POD, and GPX. As depicted in Figure 4C(a–d), yellow lines represent hydrogen bond interactions, while gray dotted lines signify hydrophobic interactions. The binding modes between pinobanksin and these enzymes are primarily hydrophobic, with binding energies of −6.3, −9.1, −8.5, and − 6.5 kcal/mol, respectively. These results indicate that pinobanksin exhibits the strongest binding affinity with the CAT enzyme, suggesting its capacity to exert antioxidant effects.

During human metabolism, highly oxidative substances like reactive oxygen species and free radicals are produced (Li & Kim, 2022). Failure to promptly eliminate these substances can result in damage to biological structures and cellular functions, contributing to aging, cardiovascular diseases, and cancer. Flavonoids have been found to influence the expression of genes, such as downregulating mutant p53 protein (Liskova et al., 2020), arresting the cell cycle, and inhibiting oncogenic enzymes, as a means to exert their anticancer effects. In this experiment, we measured the antiproliferative activity of pinobanksin on SH‐SY5Y cells (Figure 4D). The MTT assay indicated that a concentration of 250 μg/mL effectively inhibited SH‐SY5Y cell proliferation during the G1/2 phase. Hoechst staining (Figure 4D(b)) confirmed a dose‐dependent antiproliferative effect, with increasing concentration. To gain deeper insights, we investigated the impact of pinobanksin on SH‐SY5Y proteins using the WB method. Figure 4D(b) illustrates a significant effect on the expression of BAX and BCL‐2 proteins with increasing pinobanksin concentration (Abotaleb et al., 2018). BAX and BCL‐2 are part of the same protein family, regulating apoptosis by controlling mitochondrial membrane permeability. BAX dimerizes to open channels in the membrane and increase permeability, while it forms a heterodimer with BCL‐2 to decrease permeability. The BAX/BCL‐2 ratio significantly increased with rising pinobanksin concentration, confirming its potent antiproliferative activity. Likewise, the CDK4/6 complex, which plays a critical role in cell cycle regulation, showed significantly decreased expression with increasing pinobanksin concentration (Figure 4D(d)), further validating its antiproliferative capability. The cell membrane potential is crucial for normal cellular function, affecting ion exchange and maintaining the balance of internal and external substances. Pinobanksin was found to reduce the membrane potential of SH‐SY5Y cells (Figure 4D(e)), underscoring its effectiveness in inhibiting SH‐SY5Y cell proliferation.

To delve deeper into the antiproliferative effects of pinobanksin on SH‐SY5Y cells, we investigated its binding to BAX, BCL‐2, and CDK4/6 using molecular dynamics simulations. Figure 4E shows the binding modes of the BCL2–pinobanksin (Figure 4E(a)), BAX–pinobanksin (Figure 4E(b)), and CDK4/6–pinobanksin (Figure 4E(c)) complexes obtained through molecular docking. In Figure 4E(a), pinobanksin interacted hydrophobically with TYR‐67, LEU‐96, and PHE‐71 on BCL2, forming hydrogen bonds with GLU‐95 and ARG‐105, along with π‐π stack interactions with PHE‐63. For BAX–pinobanksin binding (Figure 4E(b)), BAX exhibited hydrophobic interactions with LEU‐120, LEU‐132, PRO‐88, LYS‐123, and THR‐127, as well as hydrogen bonding with THR‐127 and ASP‐84. Regarding CDK4/6–pinobanksin binding (Figure 4E(c)), pinobanksin interacted hydrophobically with LEU‐177, LYS‐68, VAL‐53, ALA‐187, LEU‐45, and ASP‐188, forming hydrogen bonds with ASP‐130, GLU‐125, and CYS‐127 on the protein. The binding energies of BCL2–pinobanksin, BAX–pinobanksin, and CDK4/6–pinobanksin were − 17.63 ± 1.08, −21.97 ± 1.38, and − 35.51 ± 0.72 kcal/mol, respectively, indicating that CDK4/6–pinobanksin displayed the strongest binding affinity. Energy decomposition revealed that van der Waals energy and electrostatic energy were the primary contributors to complex formation, followed by nonpolar nonsolvation energy. Hydrogen bonding, one of the strongest noncovalent interactions, played a crucial role in the binding. In Figure 4I, the number of hydrogen bonds formed by BCL2–pinobanksin and BAX–pinobanksin ranged from 0 to 2 during the simulation, whereas CDK4/6–pinobanksin formed between 1 and 6 hydrogen bonds. Notably, CDK4/6–pinobanksin exhibited more robust hydrogen bonding interactions during the simulation process, contributing to its stable binding and strong binding energy.

### 4.4 Pinobanksin pharmacophore screening

In Table 1 below, we present the results of our comprehensive pharmacophore database search, which yielded 21 target proteins with the highest likelihood of binding to pinobanksin. The “fitness” score in the table signifies the compatibility between the molecule and the protein, with a maximum score of 3 indicating strong compatibility. Table 4 showcases the targets scoring above 2 points, with notable performers including TNKS2, CYP1A1, and MK14, among others.

**TABLE 4:** Potential targets obtained after screening by pharmacophore database.

Molecule	Matched ligand sites	Fitness	PDB ID	Target name
Pinobanksin	A(3) A(1) D(8) R(10) R(9)	2.783	4hl5	TNKS2‐HUMAN
Pinobanksin	A(3) R(9) R(10)	2.772	4i8v	CP1A1‐HUMAN
Pinobanksin	A(3) D(8) R(9) R(10)	2.733	4eh3	MK14‐HUMAN
Pinobanksin	A(1) A(4) D(8) R(9) R(10)	2.716	5ii2	PB1‐HUMAN
Pinobanksin	A(3) A(5) R(9)	2.498	2ye4	HS90A‐HUMAN
Pinobanksin	A(1) D(8) R(9)	2.441	3iny	PNPH‐HUMAN
Pinobanksin	D(8) D(7) R(9)	2.343	1w8c	CDK2‐HUMAN
Pinobanksin	D(7) R(10) R(9)	2.306	3zrl	GSK3B‐HUMAN
Pinobanksin	A(4) D(6) R(10)	2.288	3ey4	DHI1‐HUMAN
Pinobanksin	D(7) D(6) R(10) R(9)	2.279	3bpt	HIBCH‐HUMAN
Pinobanksin	A(1) D(6) R(9)	2.275	2xfo	AOFB‐HUMAN
Pinobanksin	A(1) D(8) R(10) R(9)	2.246	2o65	PIM1‐HUMAN
Pinobanksin	D(7) R(10) R(9)	2.22	1uym	HS90B‐HUMAN
Pinobanksin	A(2) R(9) R(10)	2.209	3s8x	CAH2‐HUMAN
Pinobanksin	A(3) D(6) R(9) R(10)	2.207	4mr5	BRD2‐HUMAN
Pinobanksin	A(4) R(10) R(9)	2.17	4ie7	FTO‐HUMAN
Pinobanksin	D(8) R(10) R(9)	2.169	4epx	RASK‐HUMAN
Pinobanksin	D(8) R(9) R(10)	2.167	2ohm	BACE1‐HUMAN
Pinobanksin	D(8) R(10) R(9)	2.167	4l5m	CYH2‐HUMAN
Pinobanksin	A(3) R(9) R(10)	2.152	4i23	EGFR‐HUMAN
Pinobanksin	D(8) R(9) R(10)	2.152	2kot	S10AD‐HUMAN
Pinobanksin	A(3) R(10) R(9)	2.144	2 h96	MK08‐HUMAN
Pinobanksin	A(3) D(6) R(9)	2.141	4gv7	PARP1‐HUMAN
Pinobanksin	D(7) R(10) R(9)	2.13	3bhm	CBR1‐HUMAN
Pinobanksin	A(2) R(9) R(10)	2.122	4nbk	CASP6‐HUMAN
Pinobanksin	A(4) R(10) R(9)	2.087	4qmx	STK24‐HUMAN
Pinobanksin	A(3) D(6) R(10) R(9)	2.084	4 ft3	CHK1‐HUMAN
Pinobanksin	A(5) R(10) R(9)	2.075	3iak	PDE4D‐HUMAN
Pinobanksin	A(2) D(8) R(10)	2.074	4gqr	AMYP‐HUMAN
Pinobanksin	A(3) D(6) R(9)	2.061	2pzr	FGFR2‐HUMAN
Pinobanksin	A(2) R(9) R(10)	2.056	3v35	ALDR‐HUMAN
Pinobanksin	A(3) D(7) R(9) R(10)	2.042	4gu9	FAK1‐HUMAN
Pinobanksin	A(3) D(6) R(9)	2.034	4f1q	PAR14‐HUMAN
Pinobanksin	D(7) D(8) R(9) R(10)	2.032	1boz	DYR‐HUMAN
Pinobanksin	A(3) R(9) R(10)	2.017	2mji	FABPI‐HUMAN

The binding modes of pinobanksin with these three protein targets are further elucidated below. Subsequently, we will employ molecular docking techniques to assess the binding energy of pinobanksin with these selected proteins, shedding light on the strength and mechanisms of their interactions. We aim to delve deeper into the binding dynamics of pinobanksin with these target proteins and evaluate their potential significance in furthering our understanding of its pharmacological properties.

Molecular docking (Flores‐Romero et al., 2022) stands as a versatile technique for obtaining crucial insights into the binding energy and binding modes of small molecules with target proteins (Basu et al., 2020). In this context, we employed Vina 1.1.2 to conduct docking simulations of pinobanksin with TNKS2, CP1A1, and MK14 target proteins. Typically, docking scoring values below −6.0 kcal/mol signify strong binding affinity. The results, as shown in the table above, reveal that pinobanksin exhibits robust binding, with scores well below −6.0 kcal/mol for TNKS2, CP1A1, and MK14. This underscores the exceptional binding capability of pinobanksin with these proteins and hints at its potential efficacy as a therapeutic agent.

**FIGURE 5:**
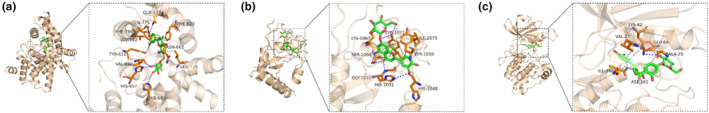
Pinobanksin pharmacophore screening.

Figure 5 depicts the binding interactions of pinobanksin, a small molecule, with CP1A1, MK14, and TNKS2 proteins. Pinobanksin exhibits strong binding affinities, primarily through hydrogen bonding and hydrophobic interactions. In Figure 5A, pinobanksin binds to the internal helix region of CP1A1, forming hydrogen bonds with residues including ARG‐455, ARG‐135, ASN‐222, ILE‐386, SER‐116, ARG‐106, THR‐385, ASP‐313, THR‐321, SER‐122, TRP‐131, ILE‐458, ALA‐317, ILE‐198, ILE‐386, VAL‐382, VAL‐322, PHE‐258, ILE‐449, and PHE‐450. Additionally, it engages in hydrophobic interactions and π‐π stacking with PHE‐123 and PHE‐224. These interactions are vital for stabilizing the complex. Figure 5B displays the binding of pinobanksin to the ATP pocket region of MK14. The interaction involves hydrogen bonding with residues such as ASP‐112, LEU‐171, ALA‐111, SER‐154, and MET‐109, as well as hydrophobic interactions with ALA‐51, LYS‐53, ASP‐112, LEU‐171, ILE‐84, and LEU‐167, contributing to the overall binding stability. Figure 5C reveals the binding of pinobanksin to the active pocket of TNKS2, characterized by hydrogen bonds with GLY‐1032, SER‐1068, TYR‐1060, and hydrophobic interactions with ILE‐1075, TYR‐1050, and TYR‐1071. Furthermore, π‐π stacking with TYR‐1071 enhances the binding affinity. These interactions are crucial for the effective binding of pinobanksin to these proteins, suggesting its potential as a therapeutic agent.

## 5 CONCLUSION AND OUTLOOK

With the continuous advancement of science and technology, there has been a notable refinement in methods for extracting active substances from plants. Innovative approaches, such as the integration of ultrasound, microwave, and enzyme‐assisted techniques, have emerged to enhance the efficiency of extracting total flavonoids from peony seed shells. These progressive methodologies align with the principles of green chemistry, emphasizing the sustainable utilization of peony seed shells as a valuable biological resource.

In the present study, we undertook a systematic investigation into the progressive purification of peony seed shell extracts through a synergistic combination of macroporous resin and C18 chromatography. This approach yielded the isolation of the pinobanksin monomer, a significant achievement with broad applicability to diverse extraction experiments. The application of this novel purification strategy has the potential to substantially elevate the purification efficiency across various contexts. Furthermore, our research delved into the biological activities of shortleaf pine, with a primary focus on its remarkable ability to inhibit SH‐SY5Y cell proliferation. Our findings revealed that shortleaf pine achieves this inhibition by interacting with crucial proteins, namely BCL‐2, BAX, and CDK4/6, elucidating the underlying molecular mechanisms. Additionally, our pharmacophore screening unveiled promising target proteins, including TNKS2, CP1A1, and MK14, which exhibit a high binding affinity for shortleaf pine. In summary, our study demonstrates the dynamic evolution of extraction techniques driven by scientific and technological progress. We have harnessed innovative methods to maximize the utilization of peony seed shells, showcasing their potential as a sustainable biological resource. Additionally, our exploration of shortleaf pine's biological activities and drug development prospects highlights the multifaceted applications of these findings in the realms of both scientific research and pharmaceutical development.

At the same time, this study also has some shortcomings. Although the method of molecular dynamics is used to verify the combination method, there are still some shortcomings between the simulation method and the real one, so the combination method still needs to be further studied. Since only in vitro experiments were used in this study, and the laboratory does not currently have the ethical review procedures required for in vivo experiments, further investigation of its pharmacophore potential is needed. In conclusion, the optimization of resource utilization in agrifood production through the extraction and utilization of bioactive compounds is a multidisciplinary endeavor that encompasses various fields, including chemistry, biology, and computational science. The integration of these disciplines offers a holistic approach to addressing the challenges of resource wastage and environmental sustainability. Our study on pinobanksin exemplifies the potential of bioactive compounds derived from agricultural by‐products to contribute to both scientific knowledge and practical applications within the agrifood industry.

## AUTHOR CONTRIBUTIONS

**Wen‐Tao Chen:** Investigation writing (equal); original draft preparation (equal); review and editing (equal). **Ying‐Yang Zhang:** Data curation (equal); writing – review and editing (equal). **Qiang Qiang:** Data curation (equal). **Lin‐Ling Zou:** Writing – original draft (equal). **Ping Zou:** Data curation (equal); writing – review and editing (equal). **Ying Xu:** Data curation (equal).

## CONFLICT OF INTEREST STATEMENT

The authors declare no conflict of interest.

## ETHICS STATEMENT

This article does not address ethical experiments, so this statement does not apply.

## SAMPLE AVAILABILITY

Samples of the compounds are available from the authors.

## INSTITUTIONAL REVIEW BOARD STATEMENT

Not applicable.

## INFORMED CONSENT STATEMENT

Not applicable.

## Data Availability

The data that support the findings of this study are available from the corresponding author upon reasonable request.
